# Empathy and perceived burden in caregivers of patients with schizophrenia spectrum disorders

**DOI:** 10.1186/s12913-021-06258-x

**Published:** 2021-03-19

**Authors:** Rosaria Di Lorenzo, Anna Girone, Nunzio Panzera, Gianluca Fiore, Margherita Pinelli, Giulia Venturi, Federica Magarini, Paola Ferri

**Affiliations:** 1 Psychiatric Intensive Treatment Facility, Mental Health and Drug Abuse Department of AUSL-Modena, Via Paul Harris, 175, 41122 Modena, Italy; 2 Casa Famiglia Querce di Mamre Centro Socio Riabilitativo Residenziale, Fondazione Casa Famiglia Mattioli Garavini- Onlus, Via Statutaria, 44, 42013 Casalgrande, Reggio Emilia Italy; 3 School of Nursing, University of Modena and Reggio Emilia, via del Pozzo, 71, 41124 Modena, Italy; 4 Resident in Psychiatry, University of Modena and Reggio Emilia, via del Pozzo, 71, 41124 Modena, Italy; 5 Department of Biomedical, Metabolic and Neural Sciences, via Campi, 287, 41125 Modena, Italy

**Keywords:** Caregiver burden, Caregiver emotional empathy, Caregiving in schizophrenia spectrum disorder patients

## Abstract

**Background:**

Caregivers of patients load different kinds of burdens, including emotional distress. Aims of this study were to evaluate both burden and empathy of caregivers who assist patients with schizophrenia spectrum disorders.

**Methods:**

We selected a sample of 60 caregivers (34 women and 26 men), who assisted patients with schizophrenia spectrum disorders treated in our local Community Mental Health Center for a 1-year minimum period. We administered two scales to our sample, Zarit Burden Interview (ZBI) and Balanced Emotional Empathy Scale (BEES), and collected data of caregivers and their assisted patients in a 3-month period. Data were statistically analyzed.

**Results:**

We reported a mean ZBI score of 49.68 (±15.03 SD) and a mean BEES score of 14.35 (±9.05 SD), indicating the perception of moderate-severe burden and low level of empathy, respectively. The analysis of internal consistency confirmed the good reliability of both ZBI (Cronbach’s alpha = 0.90) and BEES (Cronbach’s alpha = 0.77). The correlation between the two scales was not statistically significant at Spearman test. At our multiple linear regression, many variables of both caregiver and patient showed a significant correlation with the ZBI score. In particular, not living with the assisted patient and female gender of caregiver potentially decreased the burden, whereas clinical severity of assisted patient and two caregiver conditions, middle school education and spouse relationship with patient, could worsen the burden. We highlighted two positive statistically significant correlations between the total score of BEES and caregiver characteristics: being spouse and not living with assisted patient.

**Conclusions:**

Our study highlights that the caregiver burden of patients with severe psychiatric disorders is high and is associated with low emotional empathy experienced by caregivers, probably due to a defensive psychological mechanism. The conditions of spouse and cohabitation can concomitantly increase both empathy and burden in caregivers.

## Background

Relatives or close friends who provide unpaid practical daily or weekly assistance to patients are defined caregivers [1, 2]. In psychiatric practice, this role includes different tasks and responsibilities in comparison with the most well-known caregiver role of geriatric or oncological patients. Schizophrenia onset commonly occurs in early adulthood and disrupts lives of patients, who can present many social and relational disabilities, requiring caregiving for many years [3]. During the last decades, in many countries, the so-called “deinstitutionalization” has changed the primary location of health care from hospital to community-based outpatient services [4]. Nonetheless, the financial resources for community-based interventions are limited [5–7], although most severe mental disorders compromise many areas of an individual’s life, such as interpersonal relationship, work and/or self-care [8, 9]. This change in health care organization has permitted new rehabilitative programs for the patient at the cost of an increase in responsibilities for families of patients affected by severe psychiatric disorders such as schizophrenia [10]. In fact, community health care frequently involves family members as informal caregivers who play a fundamental role in the lives of individuals with schizophrenia and other serious mental illnesses [11]. The caregiver of a patient with psychotic disorders has to support the patient’s self-care and psycho-physical well-being [5, 12, 13], often providing him/her extensive support in terms of finance and housing and, at the same time, managing complex issues such as social and professional re-integration of the assisted patient. In addition, the caregiver has an important role in administering pharmacological treatment due to poor adherence to therapy often shown by patients with psychotic disorders [14–16]. Some studies showed that family caregivers of individuals with schizophrenia complain of heavier burden compared to those caregiving for an individual with a chronic medical illness [1, 17], reporting worse Health-Related Quality of Life (HRQol). Other studies reported that that emotions experienced by caregivers of patients with schizophrenia are frequently “guilt, fear and anger” due to ambivalent feelings closely related to chronic mental illness. The relationship between caregiver and patient is often so close as to be defined a dyad, which can condition the outcome of treatment. In fact, when this relationship is correct and the caregiver is well aware of his/her role, assisted patients become more adherent to treatments [18].

In accordance with some authors, family interventions were effective among people with psychiatric illness in reducing relapse risk and re-hospitalization rates [19]. On the other hand, due to the close relationship between caregiver and patient, if the caregiver experiences psychological or physical distress in caring, this condition can often lead to a worsening in the health of the assisted patient. Caregiver burden is the state resulting from necessary care tasks or restrictions that cause discomfort to the caregiver [20] due to multiple stressors linked with the caregiving activity [21]. In fact, the onerous task of the caregiver, in general, often leads to high levels of psychological pain [22, 23], such as anxiety and depression, which are the most common manifestations reported by caregivers [24, 25]. Caregivers, who are overwhelmed by these problems, tend to experience less satisfaction in their life and take less care of their health, both conditions which represent significant indicators of depression [26]. Other symptoms that can afflict caregivers are lack of sleep [27], perennial tiredness, sense of abandonment, terror and despair at the thought of facing another day of caregiving [28, 29]. Caregivers usually have limited time to maintain their social relationships, such as friendships and interpersonal activities, therefore loneliness associated with social isolation is considered a critical issue for caregivers of patients with schizophrenia and other mental health problems [30, 31]. It is also reported that uneasiness, disappointment, suffering and fear of care responsibilities are common among caregivers during the care process [30, 31].

In the last few years, an increasing number of studies have analysed the characteristics of caregivers and assisted patients in order to discover the cause of the perceived “caregiver burden”, with mixed results. Although some studies are not able to highlight any correlation between caregiver burden and patient characteristics [32–34], other research shows that some variables of patients affected by schizophrenia could worsen caregiver burden: male gender, young age, impaired functional skills, severe mental illness for a long time, multiple psychiatric hospitalizations [35–37]. Regarding condition characteristics, suicidal ideation, behavioural disorders and/or negative symptoms have the highest impact on the increase of perceived burden [38]. Regarding caregiver characteristics, some studies point out that being female, being old, having a low socio-economic status and assisting more than one patient could increase caregiver burden [36, 37, 39]. The level of caregiver empathy could affect the perceived burden.

Empathy may be defined as a complex bio-psychosocial concept which includes cognitive and emotional components, both leading to identifying with others [40]. Recent research, especially in the field of dementia, focuses on informal caregiver empathy to reduce caregivers’ depressive and anxious symptoms as well as their emotional burden, increasing caregiver and patient well-being. A study suggests that interventions for reducing caregiver depression and anxiety symptoms could be aimed at heightening cognitive empathy and lowering affective empathy [41]. Only few studies deal with this issue for caregivers of patients diagnosed with schizophrenia spectrum disorders.

The aim of the present study is to evaluate the caring burden and empathic abilities of caregivers of patients affected by schizophrenia spectrum disorders treated in a community outpatient service. We hypothesize, based on literature data, that a caregiver’s workload is inversely proportional to his/her empathic abilities and that there is an increased burden concomitant with lower levels of empathy in case of serious, chronic and greatly disabling psychiatric conditions.

## Methods

### Sample

Our sample was composed of caregivers of patients treated in a Community Mental Health Center (CMHC), in accordance with our inclusion and exclusion criteria. The researcher, before collecting data, prepared a list of patients affected by schizophrenia spectrum disorder treated in the CMHC who were assisted by a caregiver, in accordance with the indications of physicians and nurses of CMHC reported in medical records. Over the three months of data collection, the same researcher asked each consecutive caregiver reported in the list, who went to CMHC during opening hours from Monday to Saturday, to take part in the study, providing adequate information about this study. The decision of caregivers to voluntarily participate or not participate in the study was respected.

We selected caregivers in accordance with the definition of Martinez-Martin [42]: person who is not a professional caregiver, who lives with or close to the assisted patient and is directly involved in the treatment and caring of the patient’s health problem.

Inclusion criteria:

- caregivers of patients diagnosed with schizophrenia spectrum disorders according to ICD-9-CM [43] treated in local CMHC for at least 1 year; 
- caregivers and their assisted patients who provide valid informed consent to participate in the study.

Exclusion criteria:

- caregivers of patients diagnosed with other disorders or not treated in local CMHC or treated for a period less 1 than year;
- assisted patients not able to provide valid study consent due cognitive decline previously diagnosed by CMHC psychiatrists;
- caregivers and/or their assisted patients who refused valid study consent;
- caregivers involved in this study did not receive any payment for their assistance; professional caregivers, such as community mental health nurses or workers who receive a salary for giving patient assistance, were excluded.

We calculated a sample size of 145 individuals, with a margin of error of 5%, assuming a level of bilateral significance (α) of 0.05, and confidence interval of 95%, from a population of 232 patients affected by schizophrenia spectrum disorders treated in 2018 at our CMHC and assisted by non-professional caregivers.

### Design and period of the study

This observational study was aimed at evaluating the caring burden and empathy in caregivers by administering two scales: Zarit Burden Interview (ZBI) [20] and Balanced Emotional Empathy Scale (BEES) [44].

The study period of data collection and analysis lasted three months, from 21 July to 11 October 2019. The data collection period was dedicated to identification of suitable caregivers who accepted to participate in the study, and to whom the two scales were subsequently administered.

### Scales

1. Zarit Burden Interview (ZBI) [20] is a scale which can be autonomously completed, initially consisting of 29 items and currently reduced to 22 items. Each part of the scale is composed of statements which correspond to 5 preferences, ranging from 0 (never) to 4 (almost always), depending on the level of distress. Scores ranging from zero to one are evaluated as negative, while scores from two to four are regarded as positive. The ZBI has a score ranged between 0 and 88.

The ZBI 22-item version is one of the most used scales for measuring caregiver burden, which includes physical, mental, social, and economic aspects of caregiving. Originally developed to evaluate the burden of dementia patient caregivers, the ZBI has been widely applied in measuring caregiver burden of patients affected by mental illnesses. ZBI has shown good reliability and validity [45–47].

The ZBI score obtained determines four different conditions based on the severity of the emotional load:

- < 21 not present or mild burden
- 22–40 mild to moderate burden
- 41–60 moderate to severe burden
- > 60 severe burden.

The Italian version of ZBI was validated in 273 caregivers of patients with dementia [48]. We used the Italian version of ZBI which is not under license, as reported by the authors who had previously used it [46, 48].

2) Balanced Emotional Empathy Scale (BEES) is a scale used to quantify the level of emotional empathy, i.e. the degree of involvement in others’ emotions, the ability to emotionally understand the other in his/her uniqueness. The BEES was developed from the Emotional Empathic Tendency Scale and, as reported by Mehrabian (1996), who constructed the scale, the data pertaining to the process of BEES validation largely refer to the process of validation in the Emotional Empathic Tendency Scale [44]. It is composed of 30 items, of which 15 items are expressed by affirmations with positive orientation and the other 15 with negative orientation. The participants must express their degree of agreement/disagreement on a scale of 7-point Likert, with a score ranged between 0 (completely disagree) and 6 (completely agree). The BEES has been validated in the Italian version [49]. We obtained the BEES use license after purchasing the scale from Giunti Psychometrics S.r.l. Publisher.

BEES investigates the following five facets:

1. “Impermeability to the emotional feelings of others”, high scores in this dimension denote a difficulty in empathizing;
2. “Susceptibility to the emotional feelings of others”, this dimension is opposite to the previous one; in fact, high scores indicate very empathic subjects;
3. “Emotional spread responsiveness” is composed of items that are negatively oriented with respect to the construct measured; very high scores indicate the tendency to avoid emotionally moving situations, while low scores indicate individuals with a strong imagination;
4. “Susceptibility to emotional involvement with people nearby”, in which the items describe emotional situations denoted by the actual presence of the other; high scores indicate emotional contagion, on the contrary, low scores denote coldness, detachment or cruelty;
5. “Tendency to avoid emotional involvement with fragile people”, measures the specific difficulty of empathizing with the emotional experiences of the elderly and children; high scores indicate emotionally immature, self-centred individuals, while low scores indicate individuals suitable for caring for children and the elderly, even if they are handicapped or disabled.

Cronbach’s alpha of the total BEES ranged between 0.83 and 0.87 [44, 49]. In the general population, the mean total BEES score was 32±18 SD, as reported in the Italian validation study [49].

The total score of BEES indicates, if above average, individuals with high emotional empathy, who are able to respond empathically to the emotions and behaviour of others, while, if below average, it indicates individuals who have difficulty empathizing [49].

BEES has been used to evaluate empathy level in neuroscience studies [50, 51] and in different kinds of populations [49], especially among helping professions [52, 53]. In particular, BEES has been used to score the level of empathy in caregivers of patients affected by cancer [54], showing that patient’s physical pain can be correlated with caregiver’s distress. Among nursing students, BEES has been used to evaluate the effect of training on the development of empathy [55, 56].

### Modality of scale and questionnaire administration

All questionnaires were administered by the same researcher, who was not involved in the patient’s care and treatment. If caregiver decided to participate in the study, he/she was asked to sign the informed consent and the privacy form and, subsequently, ZBI and BEES were administered. Caregivers autonomously completed the two scales. Those who were not independently able to compile the scales were assisted by the researcher.

Subsequently, the same researcher filled in the form with the demographic and clinical data of the caregiver and the relative assisted patient, after having obtained the assisted patient’s consent.

### Selected variables

The following socio-demographic variables of caregivers were collected: age, gender, relationship with assisted patient (son, father, mother, etc.), schooling, work, daily time spent in caregiving.

The following demographic and clinical variables of patients assisted by our caregivers were collected: age, gender, psychiatric diagnoses in accordance with ICD-9-CM [43], medical comorbidity, substance use, period of treatment in CMHC, number of psychiatric hospitalizations and therapeutic adherence. Moreover, the scores of two evaluation scales were added: the Clinical Global Impression-Severity (CGI-S) [57], which reports the clinician assessment of illness severity on a 7-point scale, and Global Assessment of Functioning (GAF), which measures in a 0–100 point range psychosocial functioning [58]. For each patient, the variables were collected retrospectively from the medical charts and informatics system of the CMHC.

### Statistical analysis

We performed descriptive statistical variable analysis: mean and Standard Deviation (SD) for continuous variables; percentages for categorical variables. Cronbach’s alpha coefficient was used to highlight the internal consistency of both ZBI and BEES. We compared the mean scores of ZBI and BEES between the two genders through *t*-test. We correlated the two scale scores through the Spearman rank correlation test in order to assess the strength and direction of association between the two variables (empathy and burden) measured on ordinal scales such as Likert scales. We applied two separate stepwise multiple linear regressions between all selected variables and the ZBI score and total BEES score respectively in order to highlight if any variable could affect the final score of the scales and in which direction. We used the backward stepwise selection, considering variables to be removed from the model if their *p*-value was ≥0.2. We adopted the probability statistic level of significance ranging between *p* < 0.05 and two-sided alpha level of 0.05. The statistical analysis was conducted with the STATA 12 software program version (2011).

## Results

### Sample

The sample was represented by all the caregivers who agreed to participate in this study. The researcher had asked 95 caregivers but, although initially most of them had shown interest in the research, only 60 caregivers, of which 34 were women and 26 men, provided their informed consent and correctly completed the scales (response rate of 63%).

### Socio-demographic variables of caregivers

The analysis of demographic variables (Table 1) showed that our caregivers had an average age of 56.5 years, without a statistically significant difference between the two genders. Most of them graduated high school (42%), were employed (67%) and lived with the assisted subject and other people (45%), like children or their spouse. More than half of them were parents of their assisted subject (53%), without a statistically significant difference between the two genders. The caregiving time spent by our sample is 7.58 h per day, without a statistically significant difference between the two genders (Table 1).

**Table 1:** Caregiver socio-demographic variables of our sample

Variables	Male *N* = 26 (43%)	Female *N* = 34 (57%)	Total *N* = 60 (100%)
**Age, (m ± SD)**			
Years	56.69 ± 9.81	56.35 ± 15.75	56.5 ± 13.4
**Schooling, n (%)**			
Elementary school	2 (8%)	0 (0%)	2 (3%)
Middle school	4 (15%)	5 (15%)	9 (15%)
High school	12 (46%)	13 (38%)	25 (42%)
University degree	9 (34%)	15 (44%)	24 (40%)
**Work, n (%)**			
Employed	17 (65%)	23 (68%)	40 (67%)
Unemployed	2 (8%)	1 (3%)	3 (5%)
Retired for age	7 (27%)	10 (29%)	17 (28%)
Student	0 (0%)	0 (0%)	0 (0%)
**Caregiver relationship, n (%)**			
Parent	11 (42%)	21 (61%)	32 (53%)
Son/Daughter	1 (2%)	3 (9%)	4 (7%)
Husband/Wife	3 (12%)	1 (2%)	4 (7%)
Other degree of relationship	10 (38%)	10 (29%)	20 (33%)
**Home environment, n (%)**			
Living with the assisted patient	5 (19%)	10 (29%)	15 (25%)
Living with the assisted patient and others	13 (50%)	14 (41%)	27 (45%)
Not living with the assisted patient	8 (31%)	10 (29%)	18 (30%)
**Time dedicated to caregiving, (m ± SD)**			
Hours per day	7.11 ± 3.05	7.94 ± 3.25	7.58 ± 3.16

### Demographic and clinical variables of patients

As shown in Table 2, the patients assisted by our caregivers were 43.13 years old on average, without a statistically significant difference between the two genders; most of them had been suffering from a schizophrenia spectrum disorder for a long time and 90% of them had been in care at our CMHC for more than 1 year. The psychiatric disorders according to ICD-9-CM [43] suffered by the patients assisted by our sample are distributed as follows: 20% of patients were affected by schizoaffective disorder, 18% by delusional disorder, 14% by paranoid schizophrenia, 10% by disorganized schizophrenia, 7% by brief psychotic episodes, 5% by residual schizophrenia and 17% by other types of schizophrenia. Substance abuse was reported in 22% of cases, without substantial differences between the two genders. On the contrary, regarding the presence of comorbidities, there was a statistically significant difference between the two genders (Pearson chi2 = 5.21, *p* = 0.022; Table 2). From the onset of schizophrenia spectrum disorder, the totality of our sample had been hospitalized in a psychiatric ward an average of 1.8 times. Our patients presented an average score of 56.25 at GAF and an average score of 4.36 at CGI-S. Regarding their adherence to therapy, we noticed that in most cases (90%) there weren’t significant interruptions in psychiatric therapy (Table 2).

**Table 2:** Demographic and clinical variables of patients assisted by caregivers of our sample

Variables	Male N = 26 (43%)	Female N = 34 (57%)	Total N = 60 (100%)
**Age (m ± SD)**			
Years	39.83 ± 16.43	48.08 ± 16.32	43.13 ± 16.75
**Psychiatric diagnoses (ICD-9-CM), n (%)**			
Schizophrenia	21 (58%)	14 (58%)	35 (58%)
Delusional disorder	8 (22%)	3 (13%)	11 (18%)
Brief psychotic episodes	1 (3%)	3 (13%)	4 (7%)
Other types of schizophrenia	6 (17%)	4 (17%)	10 (17%)
**Medical comorbidity, n (%)**			
Absent	35 (97%)	19 (79%)	54 (90%)
Present	1 (3%)	5 (21%)	6 (10%)
**Substance use, n (%)**			
Absent	26 (72%)	21 (58%)	47 (78%)
Present	10 (28%)	3 (12%)	13 (22%)
**Period of treatment in CMHC, n (%)**			
1 year	3 (8%)	3 (13%)	6 (10%)
> 1 year	33 (92%)	21 (87%)	54 (90%)
**Psychiatric hospitalizations from schizophrenia spectrum disorder onset (m ± SD) **			
Number	1.61 ± 1.71	2.08 ± 1.50	1.8 ± 1.6
**GAF (m ± SD)**			
Total score	58.05 ± 17.24	53.29 ± 11.39	56.25 ± 15.25
**CGI-S (m ± SD)**			
Total score	4.16 ± 0.94	4.66 ± 0.48	4.36 ± 0.82
**Therapeutic adherence, n (%)**			
No therapeutic interruption	33 (92%)	21 (88%)	54 (90%)
> 1 month interruption	3 (8%)	3 (12%)	6 (10%)

### Analysis of ZBI and BEES scales

On the ZBI scale, we obtained a mean score of 49.68 (±15.03 SD), which is within the moderate-to-severe score range, without any statistically significant difference between the two genders of caregivers at *t*-test (Table 3). The alpha coefficient of Cronbach (0.90) reflects the good reliability and the internal consistency of the scale (Item-test correlation: 0.28). The BEES scale score was calculated using the correction grid provided by the authors who validated the scale in Italian [49]. On the BEES scale, we obtained a mean score of 14.35 with a standard deviation of ±9.05, indicating a low level of empathy, since, across the general population, the range of the scale varies between 7 and 56.5 [49]. The mean BEES score obtained is lower than those reported in the population for the 55–59 age range (m = 29), especially among females (m = 34.5) [49]. Cronbach’s alpha (0.77) indicates the good reliability of this scale (Item-test correlation:0.10). We did not report any statistically significant difference between the scores of two genders of caregivers at *t*-test (Table 3).

**Table 3:** ZBI and BEES scores divided by the two genders

Scales	Male N = 26 (43%)	Female N = 34 (57%)	Total N = 60 (100%)	Statistical test: ***t***-test Probability
**ZBI (m ± SD)**				
Total score	47.54 ± 14.47	51.32 ± 15.46	49.68 ± 15.03	*t* = 0.97 *p* = 0.34
**BEES (m ± SD)**				
Total score	13.58 ± 7.10	14.94 ± 10.37	14.35 ± 9.05	*t* = 0.57 *p* = 0.57
Facet 1 (F1)	5.54 ± 3.25	5.47 ± 5.05	5.5 ± 4.33	*t* = 0.06 *p* = 0.95
Facet 2 (F2)	2.58 ± 5.06	4.47 ± 4.51	3.65 ± 4.81	*t* = 1.53 *p* = 0.13
Facet 3 (F3)	4.04 ± 4.13	3.85 ± 3.08	3.93 ± 3.54	*t* = 0.20 *p* = 0.84
Facet 4 (F4)	5.85 ± 3.02	5.68 ± 3.91	5.75 ± 3.52	*t* = 0.18 *p* = 0.85
Facet 5 (F5)	−0.35 ± 3.21	−0.35 ± 3.80	−0.35 ± 3.53	*t* = 0.01 *p* = 0.99

**Fig. 1:**
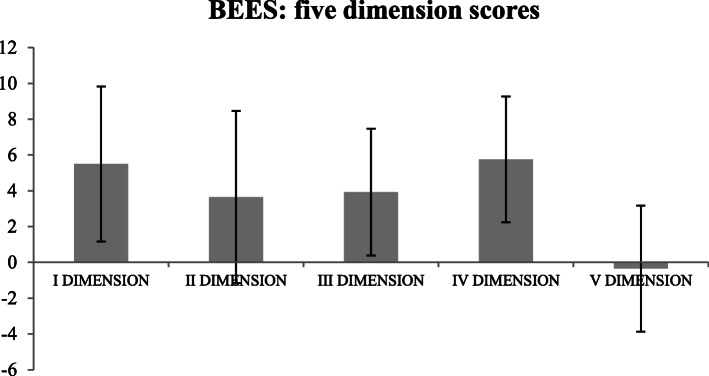
BEES: the mean scores at 5 facets

We extrapolated the mean score of the 5 facets that make up the BEES (Fig. 1):

1. “Impermeability to the emotional feelings of others”: 5.5 ± 4.33 (m ± SD)
2. “Susceptibility to the emotional feelings of others”: 3.65 ± 4.81 (m ± SD)
3. “Emotional spread responsiveness”: 3.93 ± 3.54 (m ± SD)
4. “Susceptibility to emotional involvement with people nearby”: 5.75 ± 3.52 (m ± SD)
5. “Tendency to avoid emotional involvement with fragile people”: − 0.35 ± 3.53 (m ± SD).

High scores at the first, third and fifth facet indicate a scarce capacity to empathize; on the contrary, low scores at the second and fourth facet indicate good empathic tendency.

The correlations between ZBI score and both the total BEES score and each of the five facet scores were not statistically significant (total BEES score: Spearman’s rho = 0.02; *p* = 0.88; facet 1: Spearman’s rho = − 0.22; *p* = 0.085; facet 2: Spearman’s rho = 0.16; *p* = 0.21; facet 3: Spearman’s rho = − 0.08; *p* = 0.53; facet 4: Spearman’s rho = 0.04; *p* = 0.75; facet 5: Spearman’s rho = − 0.1; *p* = 0.45).

### Variables related to ZBI and BEES scores

As shown in Table 4, at our multiple linear regression between all selected variables and ZBI score, the following variables were inversely related to the scale: the home environment, especially “not living with the assisted patient”, female gender caregiver and period of CMHC treatment; on the contrary, there was a direct correlation between ZBI score and caregiver variables (“being husband/wife” and “middle school”) as well as patient variables ("severity of schizophrenia spectrum disorders" and "patient’s age").

**Table 4:** Statistically significant variables related to ZBI score at linear multiple regression, stepwise model

Variable	Coeff.	Standard error	Probability	Confidence interval 95%
**ZBI scale total score** (R2 = 0.40; Adj R-squared = 0.27)				
**Home environment: "not living with the assisted patient"**	−11.27	5.72	*p* = 0.050	−22.73 0.23
**Patient CGI-S score**	5.92	2.19	*p* = 0.009	1.51 10.32
**Caregiver relationship: “husband/wife”**	19.9	8.82	*p* = 0.029	2.18 37.63
**Period of patient treatment in CMHC**	−0.85	0.37	*p* = 0.029	−1.6 0.09
**Caregiver gender: “female”**	−8.52	3.64	*p* = 0.024	−15.84 -1.19
**Caregiver schooling: “middle school”**	22.88	10.97	*p* = 0.042	0.81 44.94
**Assisted patient age**	0.63	0.22	*p* = 0.006	0.19 1.07

At our stepwise model of multiple linear regression between the total BEES scores (dependent variable) and all selected variables (independent ones), only two variables showed a direct statistically significant correlation with the scale: “caregiver relationship as husband/wife” and “home environment: living with the assisted patient and others” (Table 5).

**Table 5:** Statistically significant variables related to BEES score at linear multiple regression, stepwise model

Variable	Coeff.	Standard error	Probability	Confidence interval 95%
**BEES scale total score** linear multiple regression, stepwise model (R^2^ = 0.26; Adj R-squared = 0.11)				
**Caregiver relationship: "husband/wife"**	13.4	6.4	*p* = 0.042	0.52 26.27
**Home environment: "living with the assisted patient and others"**	9.6	3.56	*p* = 0.010	2.43 16.73

## Discussion

This observational study was aimed at evaluating the emotional burden and empathy among caregivers of patients affected by schizophrenia spectrum disorders, evaluated by means of two scales, ZBI and BEES, respectively. The response of our caregivers at the two scales highlights a moderate-severe emotional burden associated with low emotional empathy. Our sample of caregivers was homogeneous for sex and age: 53% of them were parents of assisted patients; 67%, despite the caring burden, were employed and 45% lived with their assisted relative in the same house, often with other relatives. Similarly, the group of assisted patients was homogeneous for demographic and clinical variables, which did not statistically significantly differ between the two genders, with the exception of medical comorbidity, more frequent in females. All patients assisted by our caregivers have been suffering from severe but stabilized schizophrenia disorders, as highlighted by CGI-S scores and low number of hospitalizations during the illness period (m = 1.8 ± 1.6 SD), respectively. Among assisted patients, therapeutic adherence was good although the global functioning was precarious, as confirmed by low scores at GAF scale. Substance abuse was reported in less than a quarter of cases.

Regarding the time dedicated to caregiving, our study highlights that caregivers spent 7.58 ± 3.16 hrs on average a day in assisting relatives; in contrast to these results, the study of Liu and colleagues [27] indicated that the management of a chronically ill patient required only 2.8 ± 2.1 hrs per day. Our different result could be explained by the great number of parents among our caregivers, who dedicated most of their time to caregiving their assisted offspring due to strong affective relationship with them.

Our sample of caregivers reported a mean ZBI score of 49.68 ± 15.03, indicating that caring burden in assisting relative patients suffering from a severe and chronic psychiatric disorder is huge. In accordance with a recent study [59], a ZBI cut-off score of 48 has been significantly predictive for identifying caregivers vulnerable to depressive and anxiety disorders.

Other studies have recently used the ZBI in psychiatric clinical practice to assess the emotional burden in caregivers of patients affected by schizophrenia spectrum disorders in many countries across the world [37], reporting high level of burden associated with schizophrenia as well as with the duration of caregiving [60, 61]. In our study, the ZBI score was significantly influenced by some characteristics of caregivers: being husband or wife of the assisted patient and middle school education were conditions of increased burden whereas being female had a protective effect. Consistently with the research of Sinha and colleagues [62], who highlighted that spouses had the highest mean burden scores in caregiving patients affected by psychosis or dementia, we found that being the husband or wife of the assisted patient can increase the caregiver burden. Another study reported that caregivers who were married, less educated, living in rural areas and with low economic income normally provided a longer period of support to their assisted individuals than others, often assuming an avoidant coping behaviour towards their assisted patients, which resulted in higher caregiver burden [37, 61, 63]. In our research, the final score of ZBI is also correlated with psychiatric disorder severity of assisted patient in a direct way and non-cohabitation with assisted patient in an indirect way. The more serious the disorder, measured by CGI-S, the more the caring burden increases proportionally; on the contrary, if caregivers do not live with their assisted patients, the caring burden is perceived to be less. This finding is in line with other studies [17, 64, 65], which put in evidence that severe and chronic disorders as well as disability conditions require complex and extensive caring, which can strongly increase the emotional load of caregivers. These results confirm other findings in the literature. Bennett and Beaudin [66] demonstrated that the caring burden statistically significantly increased in accordance with the severity of many chronic and disabling disorders: schizophrenic disorders, dementia [67, 68], stroke [69] and/or palliative care in terminally ill patients [70]. These observations indicate that different but severe health conditions can cause a huge caregiver burden, further increased if the illness persists for a long time. The correlation between the caring burden and the living environment is also highlighted in another recent investigation [71]: the caregivers who do not live with their assisted patients feel lower caring burden than those who live with their assisted individuals. Our result indicates that sharing the space and time of daily life with assisted patients is an important determinant of caregiving burden. Supportive assistance for informal caregivers is focused on caregiver empathy, which is believed to improve well-being in the caregiver and, consequently, in the recipient [41, 72, 73]. Caregivers who show higher empathy levels are considered more positive and flexible, with a better relationship with their assisted individuals, being able to experience their caregiving as a meaningful event. Caregivers who show less empathy have a less positive attitude towards caregiving [74]. Our study highlights low level of emotional empathy in our sample of caregivers, according to the total score reported at BEES (14.35 ± 9.05). Nevertheless, the score at BEES facet 5 (“Tendency not to get involved by conditions of fragile subjects”), reported by our caregivers, indicates good ability to empathize and to take care of suffering people, as already highlighted by the authors of the Italian validation of the scale [49]. This result overlaps the findings of other studies, that put in evidence the risk of reduced empathy in caregivers who have to take care of disabled patients for a long time [61, 75]. The statistically significant regression between the total BEES score and two other variables, such as being the spouse of assisted patients and living with assisted patients and others, suggest that these two conditions can increase the level of emotional empathy also in such a sample with low emotional empathy.

We hypothesize that reduced empathy capacity of our caregivers could represent a psychological defence mechanism induced by the long-term assistance to severe and chronically ill patients [75, 76].

In light of our results, we can confirm our initial hypothesis that reduced level of emotional empathy is concomitant with high emotional burden, but we cannot confirm that the two dimensions are negatively related to each other, since these two dimensions can be concomitantly increased by the caregiver’s close relationship with the assisted patient, such as being the patient’s spouse. This condition could foster both the compassion and the perceived burden, inducing ambivalent feeling in the caregiver towards the assisted patient. This result has been further supported by the correlation, highlighted by our study, between the living environment and both scales. In fact, when the caregiver lives with the assisted patient he/she could report an increase of perceived burden and if caregiver lives with the patient and other relatives the caregiver could increase his/her emotional empathy towards the patient, indicating that cohabitation with the assisted patient can increase both empathy and burden in caregivers. Among our caregivers, the parents of assisted patients showed highest interest in the scales, probably because they felt valued and wished, at the same time, to improve their caregiving. In any case, all caregivers who participated in the study manifested great emotional involvement in caring for their assisted patients and, concomitantly, complained of overwhelming “emotional burden”, thus describing two distinctive features of their activity.

### Limitations and strengths of the study

The principal limitation of the study consists of the partial representativeness of the caregivers sample, due to the small size of our sample, conditioned by the overall response rate of 60%. In fact, just over half of caregivers who were asked to participate in the study agreed to answer the scales after having given their informed consent. Our sample size was lower than that required due to the low participation rate of caregivers, which could have been conditioned by the modality of data collecting. The researcher who collected the data was not directly involved in patient care in order to avoid a collection bias and was completely unfamiliar to caregivers. For this reason, many caregivers probably did not agree to participate in the study. Some caregivers declared that the scales were too generic and not very specific in identifying subjective aspects of their daily life, showing feelings of shame in manifesting intimate information of their own lives and, indirectly, of their assisted relatives. Therefore, our sample collection may have been biased towards recruiting a reduced number of non-professional caregivers, which, in any case, represents a relevant part of informal carers who assist patients treated by our service.

Moreover, other variables, such as income and/or anxiety and depression symptoms of caregivers could have been evaluated and correlated with the scale scores. A comparison with caregivers who assisted patients affected by other psychiatric disorders could have been evaluated to deepen understanding of this topic.

Strengths of the study are represented by the homogeneity of the caregiver sample and assisted patients and in its design, which allowed us to better understand the caring burden and the empathic ability of caregivers regarding their assisted patients in a community mental health center.

## Conclusions

Our study highlights that the caregiver burden of patients with severe psychiatric disorders is similarly high to that of patients affected by medical or neurologic disorders and is associated with low emotional empathy experienced by caregivers, probably due to a psychological defense mechanism. The emotional burden could be reduced when the caregiver was a woman and did not live with the assisted patient whereas it could be increased by the clinical severity of patient disorder or by caregiver conditions, such as middle school education or being the spouse of the assisted patient. In the same way, emotional empathy could be increased when the caregiver was the spouse of the assisted patient or lived with him/her and other relatives.

Finally, we conclude that, although the perceived burden and emotional empathy did not show any statistically significant correlation between them, they could represent two different emotional responses, one negative and one positive, to the same distress condition.. Nevertheless, both dimensions are distinctive aspects of caregiving, potentially conditioned by the close relationship with the assisted patient. In the future, exploring this issue would allow us to implement interventions to safeguard the health of  the caregiver and, consequently, of the patient.

## Data Availability

The data generated and analysed in the current study are not publicly available, in order to protect the confidentiality of the study site and participants; however, further data to support the current findings can be provided by the corresponding author upon reasonable request.

## Abbreviations

HRQol: Health-Related Quality of Life
CMHC: Community Mental Health Center
ZBI: Zarit Burden Interview
BEES: Balanced Emotional Empathy Scale
ICD-9-CM: International Classification of Diseases-9th revision-Clinical modification
GAF: Global Assessment of Functioning
CGI-S: Clinical Global Impression-Severity
